# Fungi Assessment in Indoor and Outdoor Environment of Four Selected Hospitals in Peninsular Malaysia

**DOI:** 10.3390/jof11030182

**Published:** 2025-02-26

**Authors:** Nurul Izzah Ahmad, Nurul Farehah Shahrir, Anis Syuhada Omar Hamdan, Nur Amalina Kamarudin, Noraishah Mohammad Sham, Jamilah Mahmood, Yin-Hui Leong, Ratna Mohd Tap

**Affiliations:** 1 Institute for Medical Research, National Institutes of Health, Ministry of Health Malaysia, No. 1, Jalan Setia Murni U13/52, Seksyen U13, Setia Alam, Shah Alam 40170, Selangor, Malaysia; farehah.shahrir@moh.gov.my (N.F.S.); anissyuhadaomarhamdan@gmail.com (A.S.O.H.); amalina.kamarudin@moh.gov.my (N.A.K.); noraishah.ms@moh.gov.my (N.M.S.); ratna.imr@gmail.com (R.M.T.); 2 Engineering Services Division, Ministry of Health Malaysia, Level 4-7, Block E3, Parcel E, Precinct 1, Federal Government Administration Centre, Putrajaya 62590, Wilayah Persekutuan Putrajaya, Malaysia; jamilah.mahmood@gmail.com; 3 National Poison Centre of Malaysia, Universiti Sains Malaysia, Georgetown 11800, Penang, Malaysia; yhleong@usm.my

**Keywords:** fungus, indoor air, scrapped, swab, hospital environment

## Abstract

Hospital buildings require special attention to protect patients and healthcare workers from hospital-acquired infections and sick building illnesses. This is the first study to assess the prevalence of fungus in indoor air, outdoor air, and their contamination on surfaces at selected locations in four highly contaminated hospitals (A, B, C, and D) in Peninsular Malaysia. A total of 294 indoor air samples, 106 scrapped and 169 swabbed, were collected from July 2019 to August 2020. Bioaerosol concentrations were calculated using the colony-forming unit (CFU)/m^3^. Molecular identification was performed on the cultures. The internal transcribed spacer (ITS) region in the rRNA gene of the isolates was amplified by PCR. Results showed that fungal burden was in the range between 18 and 2597 CFU/m^3^. Fungal load in selected locations at Hospital D were in the higher range between 106 and 2597 CFU/m^3^, with two locations exceeding the national guidelines. Fungal genera were highly identified in air samples (47) compared to swabbed (29) and scrapped (18) samples. The dominant species were *C. halotolerans*, *C. tenuissimum*, *P. alfredii*, *P. brevicompactum*, *P. brocae*, *P. cataractarum*, and *A. aculeatus*. Fungal loads were higher in the Orthopedic and Oral Surgeon Clinic, the On Call Emergency Room, wards, and pathways.

## 1. Introduction

Microorganisms are the main causes of indoor and outdoor air pollution, which lowers indoor air quality (IAQ) and has an adverse effect on global human health [1,2]. Good IAQ is crucial for all buildings especially hospitals and healthcare settings where a wide range of ailments are treated and certain patients may emit infectious pollutants [1,3,4]. Good IAQ in hospitals is important to prevent nosocomial infection among the patients and healthcare workers. Airborne microorganisms occurring indoors are either indoor or outdoor origin [5]. The design and material of the building, the heating, ventilation and air conditioning system (HVAC), the temperature, the humidity level, the occupants, and the potential pollutant pathways are a few more elements that affect the IAQ [1,2,5]. In addition, inpatient facilities were more often contaminated with bioaerosols from people entering hospitals that cause an infestation of airborne microorganisms from outdoors. Modern mechanical ventilation systems effectively reduced their concentrations in comparison to natural ones [5].

Fungi is one of the common contaminants in indoor and outdoor environments. Indoor airborne fungal spores can cause sick building syndrome (SBS) [6,7], a condition in which the occupants especially healthcare workers and healthcare associates in hospitals experience non-specific symptoms [7] such as mucosal symptoms (eye irritations, nose catarrh and obstruction, dry and sore throat), general symptoms (headache, fatigue, nausea, dizziness, etc.), and dermal symptoms (itching and rash in the skin, face, hands or scalp) [6,8,9]. Fungi that are ubiquitous in indoor environments may cause a wide range of diseases to human especially in immunosuppressed patients with a low immunity level. Filamentous fungi such as *Aspergillus* spp., *Penicillium* spp., and *Cladosporium* spp. are common agents that can cause nosocomial infections [1,10,11,12]. *Aspergillus niger* in particular can caused invasive pulmonary aspergillosis, which is well known to develop in immunosuppressed patients including recipients of inherited or acquired immunodeficiency [13]. In addition, fungal asthma can vary from fungal-sensitized asthma to allergic broncho-pulmonary mycosis. Agarwal et al. [14] reported five challenging cases of allergic broncho-pulmonary mycosis to chronic pulmonary aspergillosis in their clinical presentation for diagnosis and treatment of various types of fungal asthma. Sivagnanasundaram et al. [15] reported that *Aspergillus* spp. can causes aspergillosis infection, which was acquired by the inhalation of airborne dust particles that carry the spores in Sri Lankan hospitals. Fungi such as yeast, *Aspergillus* spp., and *Penicillium* spp. isolated from a hospital in Iran were identified as highly pathogenic and opportunistic pathogens [10].

A number of studies have sought to assess opportunistic airborne pathogens in order to reduce hospital-acquired infections [3,16,17]. Reviews revealed that in the past two decades there have been numerous studies on indoor air quality in hospital settings around the world especially in the Asian region [2,17]. Results showed that *Aspergillus flavus*, *A. fumigatus*, and *A. niger* were identified in most hospital wards and there was evidence that the intensive care units (ICUs) and wards were home to this species when compared to other locations in the settings [1]. Hospital fungal contamination is particularly interesting since patients may be susceptible. Thus, it is essential to raise awareness of air fungal pollution in hospital indoor and outdoor areas. This study was conducted to investigate the indoor and outdoor air and surface fungal contamination of different locations from selected hospitals in Peninsular Malaysia. The results also cover the concentrations of fungus expressed in the colony-forming unit per cubic metre of air samples (CFU/m^3^). This study focused on selected highly fungus-contaminated hospitals identified by the Hospital Support Services Sector, Engineering Services Division, Ministry of Health Malaysia. The results will be helpful to improve environmental fungal contamination and reduce the health risk to individuals through the implementation of corrective measures and appropriate planning.

## 2. Materials and Methods

### 2.1. Study Sites Selection

**Figure 1:**
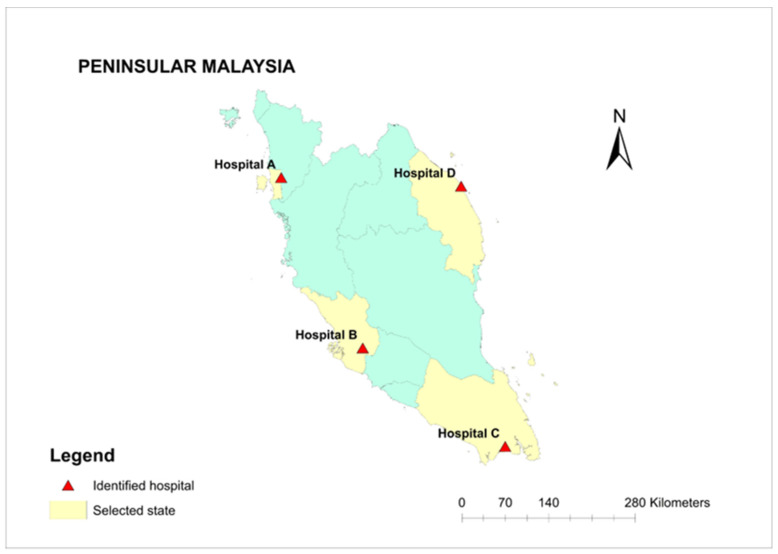
Location of studied hospitals in Peninsular Malaysia.

The Hospital Support Services Sector, Ministry of Health Malaysia (MOH) helped in identifying highly fungus-contaminated hospitals in Peninsular Malaysia. Based on their suggestion, four hospitals (Hospital A, B, C, and D) were selected based on a multistage sampling design (Figure 1), representing the following four regions in Peninsular Malaysia: the northern area (Hospital A), the west coast (Hospital B), the southern area (Hospital C), and the east coast (Hospital D). Hospital departments were divided into 4 different areas: the general areas, special services, wards, and clinics. Samples (building materials, indoor and outdoor air, swabbed samples) were collected from 2 locations of each selected general areas and clinics, and 4 locations of each special services and wards. Within the selected sampling points, the surfaces of selected utensils or furniture (the building materials) were scrapped and swabbed in the areas of visible fungal contamination, while indoor air samples were collected from all identified sampling points within the selected hospitals. This single sampling plan was conducted from July 2019 to August 2020.

### 2.2. Sampling Procedure

#### 2.2.1. Airborne Samples Collection

The Modified National Institute of Occupational Safety and Health (NIOSH) Manual and Analytical Method NMAM 0800 were used in this study. Airborne fungi samples were collected onto agar plates using a BioStage^®^ single-stage viable cascade impactor (SKC, Eighty Four, PA, USA) attached to a QuickTake^®^ 30 Sample Pump. Air samples were collected for 2 min each at a flow rate of 28.3 L/min. Air with dust particles passed through the impactor and particles were impacted onto the MEA collection medium. The plates with MEA were incubated at 25 °C for 5 days for fungal growth and counts. After 5 days, the fungal colonies were subcultured onto the Sabouraud Dextrose Agar (SDA) plates and incubated until 14 days. To avoid any interference of germs or other contaminations, the airborne sampler was sterilized before sampling using 70% ethanol and placed under a UV lamp for 20 min. The airborne sampler was then placed in a sterilized cold box and then used at the sampling location. During sampling, a 90 mm plate containing medium was placed inside the airborne sampler. The sampling pump with the representative sampler in line was calibrated every time before using a Primary Calibrator (TSI 4046, Shoreview, MN, USA) and the sampling height was measured from 1.2 to 1.5 m. The measurement was collected at the selected sampling locations for 2 min. The calculation of the colony-forming unit is in cubic metre following the formula, N = (a × 1000)/Volume where N = number of fungal CFU/m^3^ in indoor air, a = colonies counted on Petri dish (CFU), and Volume = flowrate (28.3 L/min). The number of fungi identified in the calculation was corrected to the positive hole. This is to adjust colony counts from a 400-hole impactor, for the possibility of collecting multiple particles through a hole base [18].

#### 2.2.2. Scrapped Samples Collection

The fungi was scrapped from the building materials using a sterilized stainless scalpel blade and cultured in Petri plates on the MEA medium. The plates were incubated at 25 °C for 5 days, before the fungal colonies were subcultured onto the SDA plates and incubated further until 14 days.

### 2.3. Swab Test

Sterilized pure cotton swabs were smoothly rubbed over the surface of utensils or furniture in selected locations and then pressed on Petri plates of MEA. The Petri plates were kept at 25 °C for 5 days to allow microbial development. The fungi colonies were then subcultured onto the SDA plates and incubated until day 14.

### 2.4. Fungal Identification

Using both microscopic and gross culture morphology, the cultivated fungus was identified using standard mycological procedures. According to the macroscopic and microscopic characteristics of each colony, the fungus was identified at the genus level using keys by de Hoog et al. [19]. Genus identification was confirmed by molecular identification. PCR amplification, sequencing, and BLAST analysis are identification methods as described by Mohd Tap et al. [20]. Deoxyribonucleic acid (DNA) was extracted using the ZR Fungal/Bacterial DNA MiniPrep Kit (Zymo Research (ZR), Irvine, CA, USA), which utilizes the bead beating system during the lysis step. The internal transcribed spacer 1 (ITS1) region, the internal transcribed spacer 4 (ITS4) region, and the 5.8 s ribonucleic acid (RNA) gene were amplified using a pair of universal primers ITS1 and ITS4 [21]. The ITS region has been proposed as the primary fungal barcode for species identification, as these primers are effective across many fungal species from various genera [21,22]. Amplifications were performed using MyTaq™ HS Mix (Bioline, London, UK) and were accomplished in a volume of 25 µL in the presence of 0.2 µM of each primer (ITS1 and ITS4). The polymerase chain reaction (PCR) was performed for 35 cycles with an initial denaturation step of 1 min at 95 °C in a Mastercycler Gradient (Eppendorf, Hamburg, Germany). Each cycle consisted of 15 s at 95 °C for denaturation, followed by 15 s at 56 °C for annealing and 10 s at 72 °C for the extension step. The PCR products were sent for sequencing to Apical Scientific. A nucleotide BLAST search was then performed using GenBank and a curated ISHAM Barcoding database [23,24].

### 2.5. Data Analysis

The IBM SPSS Statistics 26 was used throughout the study for statistical analysis. The data analysis was performed using the univariate descriptive statistics with frequency (n, %) and graphical representations appropriate for the nature of the data. The total fungal counts (CFU/m^3^) were compared to the Industry Code of Practice (ICOP) on Indoor Air Quality (IAQ) established by the Department of Occupational Safety and Health (DOSH), Ministry of Human Resources Malaysia in 2010 [20]. A minimum standard value of <1000 CFU/m^3^ will avoid discomfort and/or adverse health effects among occupants of an indoor or enclosed environment served by a mechanical ventilation and air conditioning system (MVAC) including an air-cooled split unit. The distribution of locations covered during the study was mapped using ArcGIS Desktop version 10.8.1.

## 3. Results

**Figure 2:**
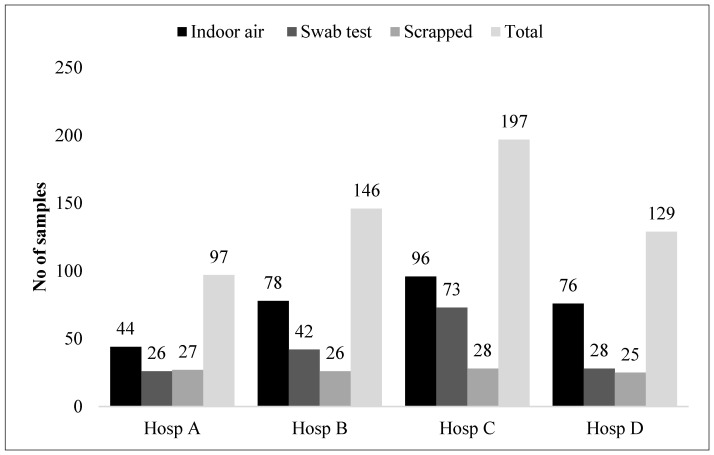
Number of samples collected using three different sampling procedures at four selected hospitals in Peninsular Malaysia.

In the present study, the air and surface fungal contamination of the selected hospitals in Peninsular Malaysia was evaluated and identified. Figure 2 shows the number of samples collected using three different sampling procedures when identifying fungus at the four selected hospitals in Peninsular Malaysia. Most samples were collected in Hospital C (197 total samples) with the highest number being indoor air (96 samples) and swab tests (73 samples) compared to the scraping of the building materials (28 samples). At Hospital B and D, the majority of samples were collected for indoor air only. For Hospital A, even indoor air samples appear to be the majority but the differences are small compared to the number of samples collected for the swabbed and scrapped samples.

### 3.1. Fungal Diversity Detected Through Different Samples

Table 1A–C lists the fungi genera collected from indoor air and scrapped and swabbed samples, respectively, from the selected hospitals. Diverse genera (47 genera) were identified in the indoor samples, followed by the swabbed samples (29 genera) and the least in the scrapped samples (18 genera). The most common genera isolated and identified in the indoor air samples were *Aspergillus* spp. (21.1%) and *Penicillium* spp. (20.1%), as well as *Cladosporium* spp. (18.7%). Other genera that were also identified at moderate numbers are *Culvularia* spp., *Phanerochaete* spp., *Talaromyces* spp., and *Pseudozyma* spp. *Penicillium* spp. were the most prevalent isolates identified in scrapped (36.8%) and swabbed (29%) samples, followed by the identification of *Cladosporium* spp. and *Aspergillus* spp. in both sample types. *Fusarium* spp. and *Parenyodontium* spp. were identified in a few number of isolates in the scrapped samples, while for the swabbed samples, two genera were the most common, namely *Culvularis* spp. and *Candida* spp.

### 3.2. Fungal Distribution at Different Hospitals

The distribution of different fungal genera isolated from four selected hospitals (A, B, C, and D) in Peninsular Malaysia (Figure 1) are shown in Table 2. A total of 68 fungal genera were isolated from all hospitals. In this study, we also identified 16 isolates of fungal endophyte. Twenty-eight further isolates were undetermined species for which there are no matches in the NCBI or Isham barcoding databases.. Results also revealed the highest diversity of fungal genera isolated were from Hospital C (40.9%), which is situated at the southern part of Peninsular Malaysia. This is followed by Hospital B (21.1%) at the central part of Peninsular Malaysia. In Hospital A and D, a number of identified isolates were comparable, 650 isolates (18.9%) and 672 isolates (19.3%), respectively.

The total number of isolates from the fungal genera from various hospitals ranges between 1 and 864 isolates (Table 2). The most abundant genera identified in the hospital environments were *Penicillium* spp. (24.8%), *Cladosporium* spp. (19.1%), *Aspergillus* spp. (18.9%), *Culvularia* spp. (4.4%), *Phanerochaete* spp. (3.1%), and *Talaromyses* spp. (3%), respectively. *Penicillium* spp. was the dominant group in Hospitals A and C, while *Aspergillus* spp. and *Cladosporium* spp. were dominant in Hospitals B and C. In contrast, Hospital D was highly contaminated with *Cladosporium* spp. and *Penicillium* spp. Four genera that were identified at more than 50 isolates within these four hospitals were *Candida* spp., *Fusarium* spp., *Parengyodontium* spp., and *Pseudozyma* spp. Another 23 genera were identified between 10 and 49 isolates while another 36 genera were identified with the least number of ≤10 isolates only.

In general, genera that were isolated from all hospitals were *Aspergillus* spp., *Cladosporium* spp., *Culvularia* spp., *Penicillium* spp., and *Talaromyces* spp. Four genera (*Hyalocladosporiella* spp., *Perenniporia* spp., and *Pleosporaceae* spp.) were only isolated from Hospital A, while eight genera (*Cryptococcus* spp., *Cystobasidium* spp., *Epicoccum* spp., *Pestalotiopsis* spp., *Phanerochaete* spp., *Pithomyces* spp., *Pseudopithomyces* spp., and *Wangiella* spp.) were only isolated from Hospital B. Another 16 and 13 fungi genera were only isolated from hospital C and D, respectively.

### 3.3. Fungal Dispersal at Different Locations in Hospitals

The distribution of fungal isolates (%) and their concentration (CFU/m^3^) in various locations of the four selected hospitals were shown in Table 3. Among all hospitals, Hospital D showed the highest number of isolates especially at the Orthopedic Specialist Clinic, the Oral Surgeon Clinic, and Ward 2B. The sites in Hospitals A and B that displayed the highest levels of fungus contamination were the Neurosurgery Ward and the On Call Emergency Room, respectively. In Hospital C, the most fungal isolates were found at the general pathway or as they called it “Laluan Menangis”. This outdoor pathway linked the Emergency and Trauma Department with the Labour Room and has been described as musty, muggy, and humid. The surrounding wall had a greenish colour and emitted a musty smell. There was substantial variation in the fungal load of the indoor air in all hospitals studied. Airborne fungal concentrations were below the limits recommended by the IAQ ICOP [20] for all locations measured across Hospital A with CFU/m^3^ for Neurosurgery Ward nearly exceeding the guidelines. The concentration ranged from 35 CFU/m^3^ to 903 CFU/m^3^. Two locations, the Oral Surgeon Clinic and the Orthopedic Specialist Clinic at Hospital D, exceeded the acceptable threshold of <1000 CFU/m^3^, while at least one location from both Hospitals B and C did. In contrast, all of the measurements for Hospital D’s total fungal counts for the indoor air were in the higher range, with three other locations showing above 500 CFU/m^3^.

**Figure 3:**
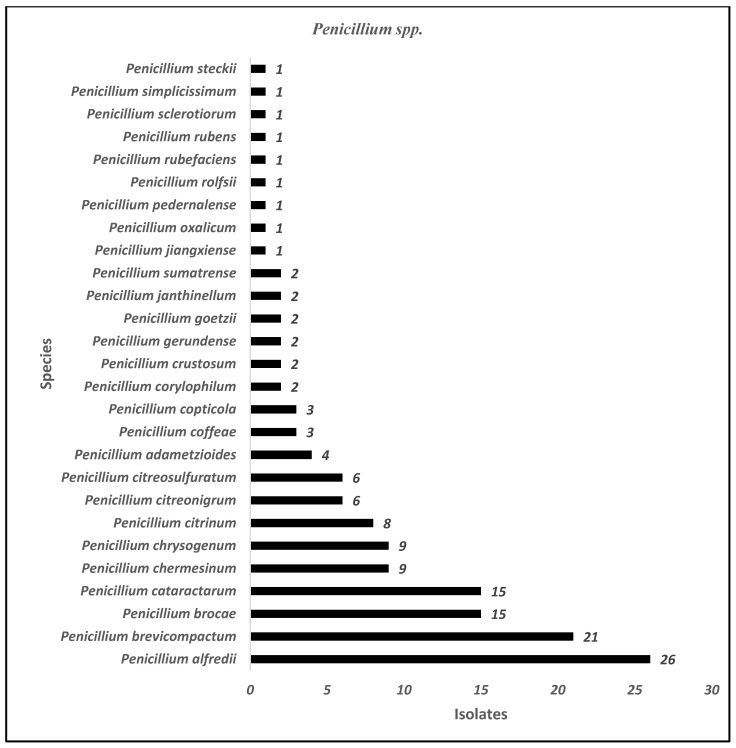
*Penicillium species* (27) identified from indoor air, outdoor air, and surface samples in four selected hospitals in Peninsular Malaysia.

**Figure 4:**
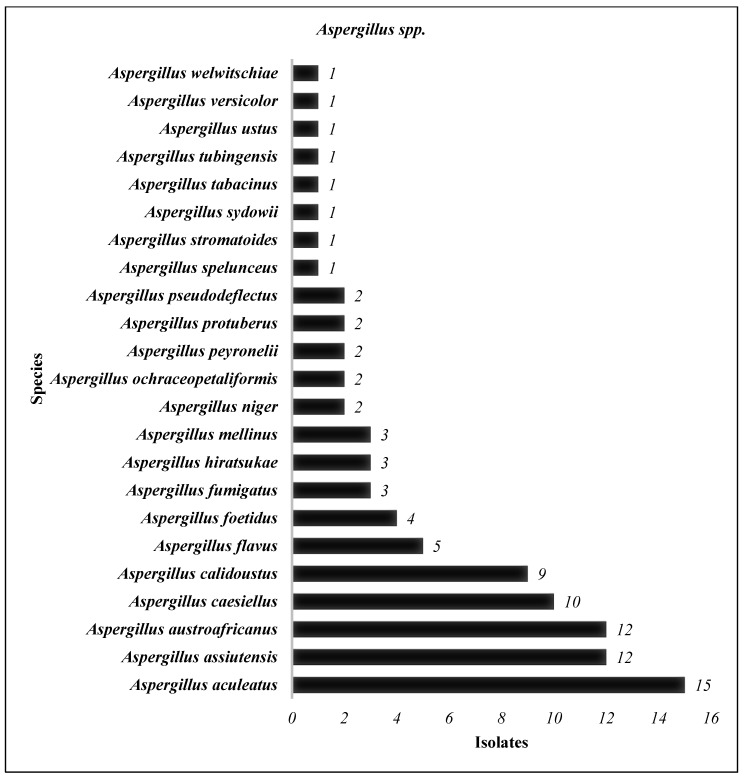
*Aspergillus species* (23) identified from indoor air, outdoor air, and surface samples in four selected hospitals in Peninsular Malaysia.

**Figure 5:**
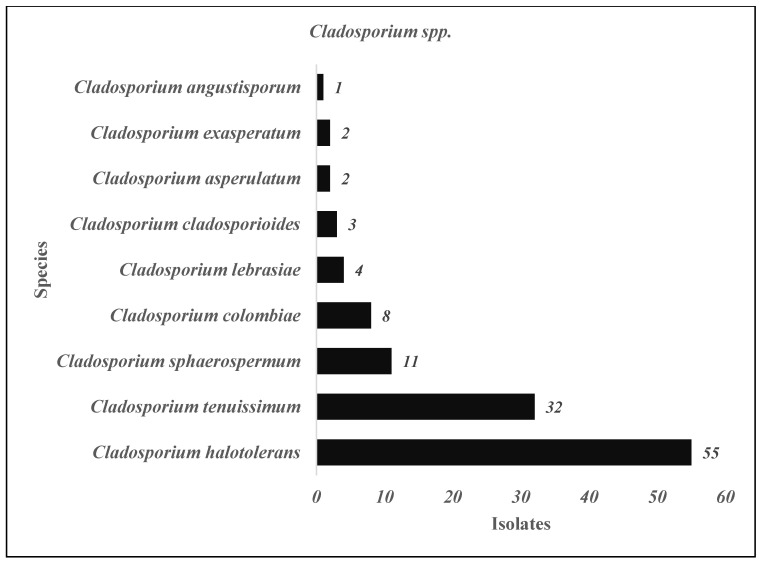
*Cladosporium species* (9) identified from indoor air, outdoor air, and surface samples in four selected hospitals in Peninsular Malaysia.

The three most prominent genera isolated from these four hospitals were *Penicillium* spp., *Aspergillus* spp., and *Cladosporium* spp. (Table 2). We identified 27 species of *Penicillium* genus in all hospitals (Figure 3), with the most frequent species identified arranged in descending order as follows: *P. alfredii* > *P. brevicompactum* > *P. brocae* and *P. cataractarum* > *P. chermesinum* and *P. chrysogenum* > *P. citrinum > P. citreonigrum* and *P. citreosulfuratum*, and so on (Figure 3). Meanwhile, of the 658 *Aspergillus* genus isolated in all studied areas (Table 2), *A. aculeatus* was the most frequent species identified (Figure 4). This is followed by a few more species of this genus that are identified in a descending manner as follows: *A. assiutensis*, *A. austroafricanus*, *A. caesiellus*, *A. calidoustus*, *A. flavus*, and *A. foetidus* (Figure 3). Although there were only nine species of *Cladosporium* identified in the study areas, the two most frequently identified were *C. halotolerans* (55 isolates) and *C. tenuissimum* (32 isolates) (Figure 5). *C. sphaerospermum* and *C. colombiae* were also identified in some study areas.

## 4. Discussion

The study was carried out to investigate the indoor and outdoor air and surfaces fungal contamination of different locations from the selected hospitals in Peninsular Malaysia. We focused on fungal diversity and their distribution throughout the selected highly fungal-contaminated hospitals. We also investigated fungal dispersal at different locations in these hospitals. Our results also covered concentrations of fungi in CFU/m^3^ of air samples and compared them to the indoor air quality for the biological contaminants guideline of the ICOP [25]. This investigation yields a number of intriguing results. Findings demonstrated a significant range in the fungal load of the indoor air across the hospitals studied. We identified 69 fungal genera and their specific species from the selected locations. We found 16 isolates of fungal endophyte and came across another 28 isolates of unidentified species. Through the application of suitable planning and corrective procedures, the outcomes will help to improve environmental fungal contamination in hospitals all over the country and subsequently reduce the health risk to individuals and occupants. Below is a discussion of each of these findings.

This study found that diverse fungi were isolated in indoor air samples (47 genera) compared to the scrapped (18 genera) or swabbed (29 genera) samples (Table 1A–C). The highest number of genera were detected from the indoor air samples compared to the other methods, namely swab samples, which has also been described in previous studies [26]. The previous studies suggested that this might be due to the influence of unidentified outside or indoor mould sources. The three most frequent and common genera isolated from several of the locations within these four hospitals were *Aspergillus* spp., *Penicillium* spp., and *Cladosporium* spp. Other genera which were also isolated frequently were *Phanerochaete* spp., *Culvularia* spp., *Talaromyces* spp., *Pseudozyma* spp., *Fusarium* spp., *Parengyodontium* spp., *Candida* spp., *Cystobasidiums* pp., and *Letendraea* spp. In line with these findings, Martinez Herrera et al. [27] reported that the most abundant fungal species in the air samples of two hospitals in Mexico City were *Penicillium* spp., *Cladosporium* spp., and *Aspergillus* spp. Similarly, the environmental surveillance of filamentous fungi in two tertiary hospitals in China found these species present in the air, on surfaces, and in tap water, with the highest fungal load during summer and spring [28]. Comparable findings were also reported in Tehran [29,30], Turkey [31], Iran [32], and Poland [28] demonstrating a consistent presence of these fungal species across diverse geographical locations. The high prevalence of *Aspergillus* spp., *Penicillium* spp., and *Cladosporium* spp. in hospital environments could be attributed to the abundance in natural environments including soil, air, and various surfaces facilitating their airborne dispersion and subsequent indoor presence [33,34,35]. These fungi are resilient and competitive, often outcompeting with others for resources [36,37]. Conducive microenvironments such as moist surfaces, ventilation systems, and the presence of organic materials combined with human activities, i.e., high foot traffic and frequent movement in and out, facilitate their infestation [33,38]. Other fungi have varying growth requirements (temperature, humidity, nutrients), and limiting spore generation and dispersion reduces colonization. These characteristics make other fungi less prevalent in hospitals settings.

Many fungal species manifested themselves around hospital environments with specific locations inside and outside the buildings comprising 27 species of *Penicillium*, 23 species of *Aspergillus*, and 9 species of *Cladosporium* (Figure 3, Figure 4 and Figure 5). Among them, the most commonly identified species from this study are as follows: seven species of *Penicillium genera* (*P. alfredii*, *P. brevicompactum*, *P. brocae*, *P. cataractarum*, *P. chermesinum*, *P. citreoningrum*, and *P. citreosulfaratum*), six species of *Aspergillus genera* (*A. aculeatus*, *A. assiutensis*, *A. austroafricanus*, *A. caesiellus*, *A. calidoustus*, and *A. flavus*), and three species of *Cladosporium genera* (*C. halotolerans*, *C. tenuissimum*, and *C. sphaerospermum*). Previous publications revealed that these several fungal species were linked to various health implications. This includes *Penicillium species*, with *P. chrysogenum* and *P. marneffei* noted for causing allergies and respiratory issues [39], with the latter capable of severe infections, particularly in immunocompromised patients [40]. *Aspergillus species*, including *A. fumigatus*, *A. flavus*, and *A. niger*, present significant concerns due to their association with aspergillosis, the production of toxic aflatoxins, and a propensity for pulmonary issues, especially in vulnerable individuals [41,42]. *Cladosporium species* such as *C. cladosporioides* and *C. herbarum* are concerning for their allergenic properties, potential to cause skin infections, and exacerbation of respiratory conditions, particularly in immunocompromised patients [43]. Monitoring for specific fungal species and keeping their levels as low as reasonably achievable is crucial. Hence, these findings underscore the importance of vigilant monitoring and control measures to mitigate the health risks associated with these fungal species in hospital environments.

Our findings revealed that the distribution of fungal colonies differs by hospital. The highest load of colonies was found in Hospital C, followed by Hospital B, Hospital A, and Hospital D. Although studies suggested that older buildings are more susceptible to fungal growth due to infrastructure issues such as leaking roofs, pipes, wall cracks, or outdated HVAC systems [44] our study found a higher prevalence of fungal growth in Hospitals C and B, which were both built in the 2000s, compared to Hospitals A and D, which were both constructed in the 1800s and 1900s, respectively. Hospital C, in particular, had known fungal issues since its construction. This aligns with findings by Park et al. [45] who reported that construction faults can lead to fungal infestations. Additionally, Kumar et al. [46] found that fungi colonized a hospital just two months after its construction, initially detected during the construction period. A study by Mahieu et al. [47] further highlighted that renovation works increased the air concentration of fungal spores in high-risk areas, but these can be mitigated using HEPA air filtration systems, which significantly reduced *Aspergillus* spp. load subsequently preventing invasive aspergillosis. No cases of invasive aspergillosis were observed during the renovation when these measures were in place, emphasizing the importance of optimal physical barriers and air filtration in decreasing airborne fungal spores in high-risk units during renovations [47].

Building defects during the post-construction phase, particularly in hospitals, are inevitable, with reported figures varying from as few as 300 defects to as high as 20,000 defects [48]. In Malaysia, newly built hospitals frequently report building defects, such as water stains on ceiling boards, indicating water seepage from roof leaks or mechanical pipework and ducting [49]. Additionally, construction quality issues related to building moisture and fungal risks include poor waterproofing and damp-proof membranes due to subpar workmanship, insufficient design, and inappropriate selection of waterproofing materials, as well as problems with sanitary systems and the use of untreated wood or mineral-based furniture [50]. Post-construction assessments described on many instances of water seepage due to torn membranes or cracked cementitious waterproofing, with installations not adhering to manufacturer specifications [48,51]. The poor installation of sanitary systems, such as untightened joints and insufficient pipe support, frequently caused leaks ranging from small drips to rare pipe bursts [52]. Untreated wooden or mineral-based furniture posed additional fungi risks due to inadequate raw material treatment, with contamination also reported [53]. Proper insulation of duct surfaces is critical to prevent condensation from air-cooled transportation, with requirements varying based on duct sizes, lengths, and pathways. Poor workmanship and a lack of quality control during construction often result in condensation and dampness, leading to fungi growth on surfaces like ceiling boards, walls, medical equipment, and furniture [50].

Mechanical ventilation design is vital for hospital buildings, with over 90% of hospital spaces incorporating air conditioning systems [48]. Imbalanced air changes, leading to stagnant air, were among many factors frequently found in hospitals. Therefore, issues related to mechanical ventilation and air conditioning must be thoroughly reviewed and inspected during post-construction assessments. Hospital design requires stringent fresh air intake ratios in specific areas such as operating theatres, clean rooms, and laboratories. Wholly fresh air is required for infection control, with the air being gradually filtered to remove microorganisms and dirt before entering the air handling unit [54]. In many cases of fungi growth found in hospitals during post-construction assessments, filtration membranes were not functioning correctly [48]. Additionally, errors in selecting fresh air intake locations, often due to last-minute decisions not specified in the design, led to intakes being placed near cooling tower areas or damp environments, inadvertently introducing fungal spores into the air conditioning system [55].

Incorporating green building architectural features, such as natural lighting, indoor vegetation, and air wells, may unintentionally foster conditions conducive to fungi growth [56]. Hospital B, with landscape gardens nearby, might introduce microbiological and particulate matter, including spores and moisture. This may be generated through indoor courtyards with water and vegetation. Air wells, initially intended for enhanced views and ventilation, may result in building dampness and air stagnation, promoting a favourable environment for fungi growth. Moreover, high turnover and a significant daily influx of individuals in hospitals further increase the likelihood of introducing fungi from external environments, subsequently colonizing vulnerable areas within the facility. Apart from that, operation factors may contribute to the differences in the prevalence of fungi colonies in different hospitals in this study. In the context of hospital construction projects in Malaysia, the typical duration ranges from 36 to 72 months [48]. However, the readiness of hospital operators, as evidenced by Hospital C, remains a persistent concern. Long-term unused spaces combined with factors conducive to fungi growth may heighten the risk of fungi contamination. Other operational factors reported to contribute to fungi growth in hospitals include underutilized spaces for prolonged periods, irregular maintenance of air conditioning systems, inexperienced maintenance teams handling new building systems, insufficient building occupancy rates leading to imbalanced air exchanges, and inadequate service personnel for building maintenance [57,58].

This study found that load of colonies varied by site. Hospital A showed the highest colony concentrations in the Neurosurgery Ward, followed by the Burn Unit and the X-ray Department. Hospital B’s highest colony concentrations were in the On Call Emergency Room, Seminar Room in Ward 6C, Record Unit, and the X-Ray Department. Hospital C had significant colonies in their general pathway and the Forensic pathway. Hospital D exhibited prevalence in almost all studied locations with four locations exhibiting the highest load of fungal counts: the Blood Collection Room, the Oral Surgeon Clinic, the Orthopedic Specialist Clinic, and Ward 2B. Two locations in Hospital D and one in both Hospitals B and C surpassed the ICOP Guidelines. The differences in the presence of colonies by site were reported in other studies as well [27,59]. The findings are alarming, particularly as certain areas, such as the Neurosurgery Ward, X-ray Department, and Pediatric Ward, where critically ill and immunocompromised patients are often located, are susceptible to opportunistic and fungal infections, which are associated with high mortality and morbidity rates. Moreover, in some hospital settings, fungal prevalence is notably observed in pathways and seminar rooms, posing a risk to staff and potentially contributing to respiratory illness, sick building syndrome, and leading to increased absenteeism, illness, and healthcare costs. Corridors and pathways with high patient traffic could reintroduce fungal spores into wards, further spreading contamination. In addition, the prevalence of fungal contamination in the Cytology Laboratory is concerning, as it can compromise laboratory results and potentially lead to incorrect diagnoses and treatments due to contaminated cytological samples, resulting in diagnostic inaccuracies or delays [60,61,62]. Although the presence of fungal colonies is less prominent in other settings, such as the Nuclear Department, Rehabilitation Medicine Department, Labour Room (injection room), pantry, store, and the Administrative Unit, it still presents significant risks [12,63]. In high-risk and immunocompromised patients, the development of invasive aspergillosis can occur even with low concentrations of *Aspergillus* spp. (less than 1 CFU/m^3^) [63]. Moreover, despite the lack of consensus on defining safe fungal spore levels, previous research indicates wide variations in airborne fungal contamination levels during hospital outbreaks, ranging from 0 to over 100 spores/m^3^ [63]. Thus, more stringent measures are required to address these concerns.

Hospital fungal infestation prevention calls for a multipronged strategy. It is advised that incorrect hospital construction be avoided by performing routine building maintenance and taking care of structural flaws such moisture buildup and leaks. Using high-quality building materials resistant to moisture and fungi, along with safe fungicidal treatments, enhances prevention. Improvements in equipment installation and design should be made for hospitals that have air treatment systems in place. Incorporating air filtration systems and dehumidifiers maintains optimal indoor air quality. Regular HVAC system inspections help prevent fungal spore spread and adequate air ventilation systems help mitigate dampness in buildings under construction. The use of drying equipment, such as dehumidifiers and air blowers for reverse airflow, has proven effective in reducing the risk of fungal growth during construction and is beneficial during operation.

Hospital managers attempt to quantitively and qualitatively evaluate the indoor air of hospitals periodically. These inclusive implementing stringent infection control protocols, especially in high-risk areas, which mitigates fungal contamination. Regular infection control risk assessments, particularly during construction, ensure appropriate precautions are taken. Future studies should identify fungal presence cut-off levels for critical areas like ICUs and neonatal units. All these measures are essential to ensure preventing patient morbidity and mortality from fungal infection in the hospital.

## 5. Conclusions and Recommendations

Fungal occurrence and species distribution in the indoor air varied between different hospital buildings and their departments. Hospital D showed the highest number of isolates at three selected locations with two of the readings exceeding the acceptable threshold of <1000 CFU/m^3^. Moreover, at least one location from both Hospitals B and C exceeded the ICOP (2010) Guidelines. The results also revealed that all of the measurements for total fungal counts in indoor air for Hospital D were in the higher range of >200 CFU/m^3^. The Orthopedic Specialist Clinic, the Oral Surgeon Clinic, Ward 2B, the Neurosurgery Ward, the On Call Emergency Room, and the general pathway are determined to have a higher fungal load. This study also revealed that fungal genera were highly identified in air samples compared to scrapped and swabbed samples. The most identified genus throughout this study were *Penicillium* spp. > *Cladosporium* spp. > *Aspergillus* spp. > *Curvularia* spp. > *Parengyodontium* spp. > *Phanerochaete* spp. > *Talaromyces* spp. > *Fusarium* spp. The most dominant species in the study locations were *C. halotolerans*, *C. tenuissimum*, *C sphaerospermum*, *P. alfredii*, *P. brevicompactum*, *P. brocae*, *P. cataractarum*, and *A. aculeatus.*

This study also faces some limitations. Since this is a descriptive study, causality was unable to be established. Future studies are needed to incorporate healthcare-associated infections to better assess this alarming issue. In addition, we were unable to account for the various factors that could potentially confound the difference in the prevalence of fungi in different hospital settings, such as seasonality, weather, and the ventilation system, which could influence the spore levels of airborne fungi. Despite these limitations, this study highlights the numerous fungal species contaminating hospitals across Malaysia, providing invaluable information for future research and policy development. Moreover, multiple methods were used in this study, including scraping, indoor air sampling, and swabbing, each with distinct advantages and limitations. Additionally, fungal species were identified through molecular methods involving DNA extraction, PCR amplification, and sequencing. The combination of these approaches ensured a comprehensive assessment of fungal presence in the hospital environments studied.

## Figures and Tables

**Table 1:** (**A**) Fungi genera collected in indoor air and outdoor samples from four selected hospitals in Peninsular Malaysia. (**B**) Fungi genera collected from scrapped samples from four selected hospitals in Peninsular Malaysia. (**C**) Fungi genera collected from swab test samples from four selected hospitals in Peninsular Malaysia.

**A**			
**No**	***Genera***	**Isolates**	**%**
1	*Aspergillus*	62	21.1
2	*Candida*	4	1.4
3	*Choanephora*	1	0.3
4	*Cladosporium*	55	18.7
5	*Cochliobolus*	1	0.3
6	*Cryptococcus*	1	0.3
7	*Curvularia*	11	3.7
8	*Diaporthe*	2	0.7
9	*Dirkmeia*	3	1.0
10	*Duportella*	1	0.3
11	*Epicoccum*	1	0.3
12	*Fusarium*	3	1.0
13	*Hyalocladosporiella*	1	0.3
14	*Letendraea*	1	0.3
15	*Lichtheimia*	1	0.3
16	*Meyerozyma*	1	0.3
17	*Microdochium*	1	0.3
18	*Moesziomyces*	4	1.4
19	*Mucor*	5	1.7
20	*Neopestalotiopsis*	1	0.3
21	*Nigrospora*	2	0.7
22	*Parengyodontium*	4	1.4
23	*Penicillium*	59	20.1
24	*Peniophora*	2	0.7
25	*Perenniporia*	2	0.7
26	*Periconia*	2	0.7
27	*Phaeosphaeria*	1	0.3
28	*Phaeosphaeriopsis*	1	0.3
29	*Phanerochaete*	13	4.4
30	*Phellinus*	1	0.3
31	*Phlebiopsis*	4	1.4
32	*Pleosporaceae*	1	0.3
33	*Pleosporales*	4	1.4
34	*Pseudopestalotiopsis*	2	0.7
35	*Pseudozyma*	8	2.7
36	*Psilocybe*	1	0.3
37	*Pyrenochaetopsis*	1	0.3
38	*Rhizopus*	1	0.3
39	*Rhodosporidium*	1	0.3
40	*Rhodotorula*	1	0.3
41	*Simplicillium*	1	0.3
42	*Strophariaceae*	1	0.3
43	*Talaromyces*	10	3.4
44	*Trichoderma*	1	0.3
45	*Ustilago*	1	0.3
46	*Vesiculozygosporium*	1	0.3
47	*Wardomyces*	1	0.3
**B**			
**No**	***Genera***	**Isolates**	**%**
1	*Aspergillus*	12	11.3
2	*Cladosporium*	27	25.5
3	*Cryptococcus*	1	0.9
4	*Curvularia*	2	1.9
5	*Didymella*	1	0.9
6	*Ectophoma*	1	0.9
7	*Fusarium*	6	5.7
8	*Lecanicillium*	1	0.9
9	*Paramyrothecium*	1	0.9
10	*Parengyodontium*	6	5.7
11	*Penicillium*	39	36.8
12	*Phialophora*	1	0.9
13	*Phomopsis*	1	0.9
14	*Rasamsonia*	1	0.9
15	*Simplicillium*	2	1.9
16	*Stachybotrys*	1	0.9
17	*Trichoderma*	1	0.9
18	*Wangiella*	1	0.9
**C**			
**No**	***Genera***	**Isolates**	**%**
1	*Acremonium*	1	0.6
2	*Aspergillus*	20	11.8
3	*Aureobasidium*	2	1.2
4	*Candida*	5	3.0
5	*Cladosporium*	39	23.1
6	*Cochliobolus*	2	1.2
7	*Curvularia*	6	3.6
8	*Cystobasidium*	1	0.6
9	*Fusarium*	2	1.2
10	*Hypocreales*	1	0.6
11	*Letendraea*	5	3.0
12	*Montagnula*	1	0.6
13	*Myrothecium*	1	0.6
14	*Nigrospora*	2	1.2
15	*Paraphaeosphaeria*	1	0.6
16	*Parengyodontium*	4	2.4
17	*Penicillium*	49	29.0
18	*Periconia*	1	0.6
19	*Pestalotiopsis*	1	0.6
20	*Pithomyces*	1	0.6
21	*Pseudopestalotiopsis*	3	1.8
22	*Pseudopithomyces*	1	0.6
23	*Pseudozyma*	1	0.6
24	*Rhizopus*	1	0.6
25	*Rhodotorula*	1	0.6
26	*Stachybotrys*	2	1.2
27	*Starmerella*	1	0.6
28	*Syncephalastrum*	1	0.6
29	*Talaromyces*	2	1.2

**Table 2:** Distribution of different fungal genera isolated from four selected hospitals in Peninsular Malaysia.

No	*Genera*	Hospital A	Hospital B	Hospital C	Hospital D	Total
1	*Acremonium*				3	3
2	*Aspergillus*	177	196	21	67	658
3	*Aureobasidium*			20		20
4	*Candida*		7	39	19	65
5	*Choanephora*			10		10
6	*Cladosporium*	54	196	235	181	666
7	*Cochliobolus*	6		16		22
8	*Cryptococcus*		4			4
9	*Curvularia*	50	26	59	17	152
10	*Cystobasidium*		7			7
11	*Diaporthe*				13	13
12	*Didymella*			4		4
13	*Dirkmeia*			29		29
14	*Duportella*			10		10
15	*Ectophoma*			4		4
16	*Epicoccum*		7			7
17	*Fusarium*		11	48	8	67
18	*Hyalocladosporiella*	14				14
19	*Hypocreales*				1	1
20	*Lecanicillium*			4		4
21	*Letendraea*		6	30		36
22	*Lichtheimia*			14		14
23	*Meyerozyma*				11	11
24	*Microdochium*				7	7
25	*Moesziomyces*		7	10		17
26	*Montagnula*			6		6
27	*Mucor*		14	26		40
28	*Myrothecium*			9		9
29	*Neopestalotiopsis*				11	11
30	*Nigrospora*			26		26
31	*Paramyrothecium*			1		1
32	*Paraphaeosphaeria*			5		5
33	*Parengyodontium*	23		19	8	50
34	*Penicillium*	285	187	253	139	864
35	*Peniophora*				13	13
36	*Perenniporia*	14				14
37	*Periconia*			16	4	20
38	*Pestalotiopsis*		6			6
39	*Phaeosphaeria*				8	8
40	*Phaeosphaeriopsis*				8	8
41	*Phanerochaete*			75	32	107
42	*Phellinus*			14		14
43	*Phialophora*				2	2
44	*Phlebiopsis*			24	17	41
45	*Phomopsis*				3	3
46	*Pithomyces*		6			6
47	*Pleosporaceae*	7				7
48	*Pleosporales*				33	33
49	*Pseudopestalotiopsis*			32		32
50	*Pseudopithomyces*		6			6
51	*Pseudozyma*		4	50	10	64
52	*Psilocybe*				6	6
53	*Pyrenochaetopsis*				8	8
54	*Rasamsonia*				4	4
55	*Rhizopus*		6	2		8
56	*Rhodosporidium*				4	4
57	*Rhodotorula*				14	14
58	*Simplicillium*	7			5	12
59	*Stachybotrys*				7	7
60	*Starmerella*			7		7
61	*Strophariaceae*				7	7
62	*Syncephalastrum*			5		5
63	*Talaromyces*	13	22	66	2	103
64	*Trichoderma*			8		8
65	*Ustilago*		3	7		10
66	*Vesiculozygosporium*			10		10
67	*Wangiella*		2			2
68	*Wardomyces*			11		11
	**Total**	**650**	**723**	**1422**	**672**	**3467**
	**Unidentified genera**					
1	Fungal endophyte				16	16
2	Unidentified species		13	12	3	28
	**Total**		**13**	**12**	**19**	**44**
	**Grand Total**	**650**	**732**	**1434**	**691**	**3511**

Fungal endophyte—identified by NCBI database but not mentioned in Isham Barcoding. Unidentified species—no identification found from NCBI and Isham Barcoding databases.

**Table 3:** Distribution of isolates (%) and concentration (CFU/m^3^) of fungal in indoor air and outdoor samples from four hospitals at different locations in Peninsular Malaysia.

No	Hospital/Locations	Isolates (%)	CFU/m^3^
Hospital A		116 (11.7%)	
1	Pharmacy	8	143
2	Emergency Department	16	288
3	Nuclear Department	2	35
4	X-ray Department	18	325
5	Burn Unit	24	438
6	Neurosurgery Ward	48	903
Hospital B		284 (28.6%)	
1	Pharmacy	6	106
2	Histopathology Laboratory	11	198
3	ENT Clinic	22	399
4	On Call Emergency Room	105	2152
5	Oral Surgery Department	18	325
6	Rehabilitation Medicine Department	5	88
7	Account Unit	12	216
8	Record Unit	27	495
9	Pediatric Ward	17	307
10	Seminar Room in Ward 6C	42	784
11	Pantry in Ward 6D	NA	NA
12	X-ray Department	19	345
Hospital C		197 (19.8%)	
1	Examination Room	11	198
2	Cafe	13	233
3	Labour Room—Injection Room	4	71
4	Pathway between Medical Store and Pharmacy	18	325
5	Forensic Pathway	22	399
6	General Pathway	66	1274
7	Emergency Exit	17	307
8	Pantry for Dental and Pediatric Clinic	1	18
9	Praying Room for Ladies	17	307
10	Store in Haemodialysis Unit	5	88
11	Administrative Unit	2	35
12	Pediatric Ward	15	270
13	Yellow Zone for Emergency Department	6	106
Hospital D		396 (39.9%)	
1	Blood Collection Room	38	705
2	Emergency and Trauma Department	11	198
3	Oral Surgeon Clinic	53	1005
4	Orthopedic Specialist Clinic	123	2597
5	Mother and Child Treatment Centre Lobby	14	253
6	Cytology Laboratory	17	307
7	O&G Department	21	382
8	Daily Pediatric Treatment Centre	20	362
9	Ward 4A	6	106
10	Ward 7AB	28	512
11	Ward 2B	46	864
12	Ward 3A	19	345
Total		993	

ICOP (2010): the acceptable limits for total fungus counts < 1000 CFU/m^3^.

## Data Availability

The original contributions presented in the study are included in the article, further inquiries can be directed to the corresponding author.
